# Anthraquinone-based turn-on fluorescence probe for selective and sensitive detection of Cu^2+^ ions

**DOI:** 10.3389/fchem.2026.1761329

**Published:** 2026-04-14

**Authors:** Jiang-song Jia, Hong-lei Li, Yi-fan Xu, Wen-ming Zhao, Jun Sun

**Affiliations:** 1 Department of Pharmacy, Henan Provincial People’s Hospital; People’s Hospital of Zhengzhou University; People’s Hospital of Henan University, Zhengzhou, China; 2 Department of Pharmacy, Kangda College of Nanjing Medical University, Lianyungang, China

**Keywords:** 9,10-anthraquinone, copper, probe, Schiff base, turn-on

## Abstract

**Introduction:**

A novel anthraquinone-hydrazone fluorescent probe (AFSA) was designed and synthesized for the selective and sensitive detection of Cu^2+^ ions, which is of great significance for environmental heavy metal monitoring.

**Methods:**

The sensing performance of AFSA was systematically investigated in an EtOH/H_2_O (5:1, V/V) binary solution system. Its molecular structure was characterized using multiple spectroscopic techniques, and theoretical spectral properties were further calculated via the GaussView 9.0 quantum chemical program. The binding stoichiometry between AFSA and Cu^2+^ was determined using Job’s continuous variation method.

**Results:**

AFSA exhibited a distinct turn-on fluorescence response toward Cu^2+^, with a detection limit of 0.86 μM at 766 nm and a response time of less than 1 min. The fluorescence emission remained stable over a wide pH range of 5.0–11.0. Selectivity assays demonstrated negligible interference from common coexisting metal ions, including Al^3+^, Ba^2+^, Ca^2+^, Co^2+^, Hg^2+^, Fe^3+^, K^+^, Mn^2+^, Na^+^, Ni^2+^, Pb^2+^, and Zn^2+^.

**Discussion:**

The above results indicate that AFSA is a promising fluorescent probe for Cu^2+^ detection with excellent selectivity, high sensitivity and favorable practical applicability. This work provides a feasible molecular design strategy and a reliable anthraquinone-hydrazone scaffold for the development of high-performance chemosensors toward environmental heavy metal ion monitoring.

## 1 Introduction

Copper (Cu^2+^), one of the most prevalent metals in the earth’s crust, is an essential trace metal for sustaining normal physiological functions in living organisms. It participates in multiple vital biological processes invluding iron metabolism, erythropoiesis, copper-containing enzymes, and the proper operation of the body’s hematological systems (Chellan and Sadler, 2015; Chen et al., 2024; Liang et al., 2024; Zhu et al., 2024). However, with the widespread application of copper in industry, agriculture, and daily human acricities, excessive Cu^2+^ has become a major environmental pollutant. Because of the significant health hazards associated with copper ion pollutant, posting severe threats to ecological systems and human health. To safeguard public health,the World Health Organization (WHO) has established a strict limit of 20 μM for Cu^2+^ in drinking water (Zhang et al., 2022; Zhou et al., 2022). Both deficiency and overload of Cu^2+^ can trigger a series of health disorders: free Cu^2+^, formed by the dissociation of copper-albumin complexes and other small molecule chelates in organisms, is associated with the progression of anemia, Parkinson’s disease, Alzheimer’s disease, and other serious illnesses (Brewer, 2010; Li et al., 2024; Quintanar, 2024; Teschke and Eickhoff, 2024). Thus, the accurate and sensitive detection of Cu^2+^ is of great significance for analytical chemistry, environmental monitoring, and biomedical research (Albertos et al., 2022; Dujols et al., 1997; Uauy et al., 1998).

Various analytical methods have developed for Cu^2+^ detection, such as electrochemical analysis, spectroscopic approaches, biochemical tests and ions exchange chromatography (Gumpu et al., 2015; Moghaddam et al., 2019). Nevertheless, these conventional approaches suffer from inherent drawbacks, including the requirement for bulky and expensive equipment, tedious operational procedures, and high technical proficiency of operators. These limitations make them costly and impractical for on-site and real-time monitoring of Cu^2+^ in complex samples. In recent years, fluorescent sensing has emerged as a promising alternative for ion detection, owing to its outstanding advantages of high selectivity, rapid response, exceptional sensitivity,and simple operation (Ji et al., 2022; Mashhadizadeh et al., 2006; Udhayakumari et al., 2014; Zeng et al., 2021). Our earlier research, successfully designed a Schiff base fluorescent sensor with rapid response and high specificity for Cu^2+^ detection (Liu et al., 2023). Consequently, developing probes with enhanced selectivity and sensitivity for Cu^2+^ recognition remains an urgent priority.

Fluorescence probes based on Schiff base, known for their hard-base donor sites featuring N–O-rich coordination environments, demonstrate improved structural stability and exhibit both visual recognition and fluorescence detection (Alam and Khan, 2023; Kumari et al., 2023). The presence of conjugated double bonds (-C=N-) in these probes enables structural isomerization, speeding up the non-radiative transition of the Schiff base in its excited state, leading to diminished fluorescence emission. The introduction of metal ions results in the formation of complexes with the Schiff base probes, causing the extraction of protons from the donor sites. This interaction hampers the structural isomerization of -C=N- and suppresses the excited-state proton transfer process, accompanied by noticeable alterations in color and fluorescence characteristic (Golbedaghi et al., 2021; Ma et al., 2025). Moreover, Schiff bases offer inherent advantages including higher synthetic yield, robust structural stability, superior photophysical properties, and enhanced fluorescence performance, which lay a solid foundation for their development as high-performance chemosensors.

Anthraquinone and its derivatives are a class of promising organic fluorophoress that have attracted persistent research interest owing to their excellent chromogenic and fluorogenic properties (Kar et al., 2007; Wang et al., 2023; Zhu et al., 2022). These molecules exhibit unique merits such as high molar absorption coefficients, characteristic absorption and emission peaks in the visible spectral region, direct visual detectability, and low biological toxicity, making them ideal building blocks for the construction of chemical sensing platforms. Consequently, anthraquinone derivatives have been widely utilized as versatile platforms for linking with ionophores that exhibit selective recognition abilities for both anions and cations. Zhao et al.synthesized a versatile anthraquinone-based Schiff base probe DMHB via the condensation of anthraquinone derivatives with salicyl hydrazide; this probe exhibited excellent performance in pH monitoring and Cu^2+^/S^2−^ dual-ion detection, and showed great potential for bio-imaging applications, which fully demonstrated the applicability of anthraquinone derivatives in advanced sensing technologies (Zhao et al., 2025). In this work, a novel anthraquinone-Schiff base fluorescent probe AFSA was designed and synthesized for the selective detection of Cu^2+^. AFSA integrates anthraquinone as the fluorophore and salicylaldehyde hydrazone Schiff base as the ion recognition group, forming a rigid conjugated molecular structure that effectively reduces non-radiative energy loss and improves fluorescence quantum yield of the probe. In an EtOH/H_2_O binary solvent system, AFSA exhibits high sensitivity toward Cu^2+^ with the advantages of rapid response, low cost, and an ultralow detection limit.

**SCHEME 1:**
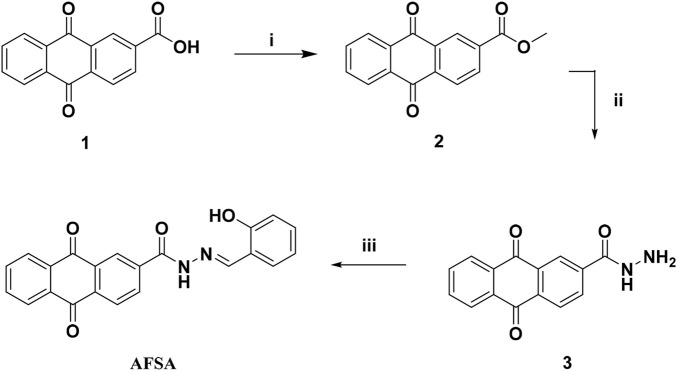
Synthesis route to probe AFSA. i) SOCl_2_, 78 °C, 2 h; MeOH, rt, overnight, yielding 92.01%; ii) 85% hydrazine hydrate, EtOH,rt, overnight, yielding 82.55%; iii) salicylaldehyde, EtOH, acetic acid, 4 h, yielding 61.82%.

The synthesis of AFSA is deicted in Scheme 1, and the light-induced electron transfer (PET) mechanism underlying the Cu^2+^ recognition process of AFSA was proposed and verified via Job’s plot analysis. Notably, different from most reported anthraquinone-based probes that exhibit a “turn-off” fluorescence response to metal ions, AFSA shows a rare “turn-on” fluorescence response toward Cu^2+^ with a near-infrared emission peak at 766 nm,which significantly reduces background interference in practical detection and realizes specific and sensitive identification of the target ion. Furthermore, AFSA possesses a broad pH working range (5.0–11.0) and an ultra-fast response time (<1 min), which is markedly superior to most similar Schiff base probes that suffer from narrow pH adaptability or slow response kinetics, thus endowing AFSA with excellent practical application potential in complex natural water and environmental samples. These findings not only highlight the unique structural and performance advantages of the AFSA probe but also further confirm the great application potential of anthraquinone-Schiff base derivatives in the development of advanced Cu^2+^ chemosensors, which provides a new design strategy for fluorescent probes applied in analytical chemistry and environmental monitoring.

## 2 Results and discussions

### 2.1 Chemistry

Compound AFSA was synthesized following the procedure outlined in Scheme 1. Initially, compound 1 was reacted with SOCl_2_ and stirred at 78 °C for 2 h. After removing the excess SOCl_2_, an excess of methanol was added to the residue, and the mixture was stirred for an additional 6 h. The resulting solid was collected by precipitation, dried, and yielded 9,10-anthraquinone-2-carboxylate (2). Next, compound 2 was mixed with 85% hydrazine hydrate in ethanol and stirred overnight at room temperature. The mixture was then poured into crushed ice, and the precipitate was filtered to obtain 9,10-anthraquinone-2-carbohydrazide (3). Finally, compound 3 and salicylaldehyde were dissolved in ethanol with two drops of acetic acid as a catalyst. The solution was refluxed at 78 °C for 4 h, after which the precipitated solid was filtered and washed with cold ethanol to yield the target compound, AFSA. All synthesized compounds were characterized using elementary analysis and spectroscopic techniques, confirming their structural integrity and purity.

The facile three-step synthetic strategy with high intermediate yields and simple purification processes endows AFSA with potential for scale-up preparation, laying a solid experimental foundation for its subsequent practical application in Cu^2+^ detection.

### 2.2 Characterization of probe

#### 2.2.1 Fluorescence spectra of probe AFSA for selectivity and anti-interference detection

**FIGURE 1:**
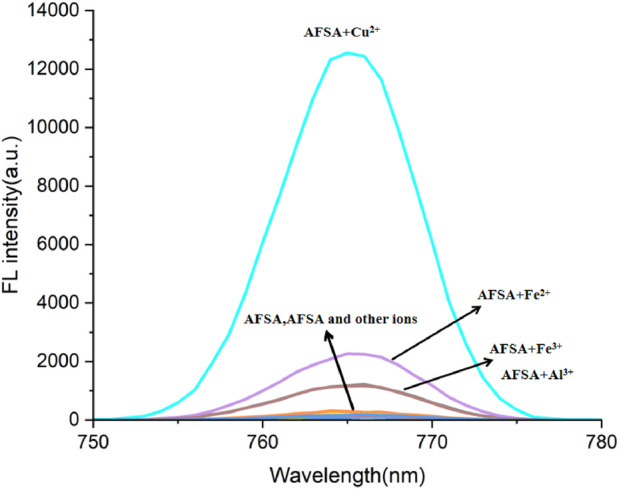
Fluorescence spectra of AFSA (20 μM) with various metal ions (K^+^, Ba^2+^, Cd^2+^, Hg^2+^, Fe^2+^, Hg^2+^, Mg^2+^, Pb^2+^,Mn^2+^, Na^+^, Ni^2+^, Fe^3+^, Co^2+^, Al^3+^, Cu^2+^, and Zn^2+^) (10 μM) in EtOH/H_2_O(V/V = 5:1) (λ_ex_ = 384 nm, λ_em_ = 766 nm).

The fluorescence response of AFSA probe to Cu^2+^ was studied in a mixed EtOH and water solution (V/V = 5:1), as depicted in Figure 1. Before assessing AFSA’s selectivity, various metal ions (K^+^, Ba^2+^, Cd^2+^, Hg^2+^, Fe^2+^, Mg^2+^, Pb^2+^, Mn^2+^, Na^+^, Ni^2+^, Fe^3+^, Co^2+^, Al^3+^, Cu^2+^, and Zn^2+^) were examined. Each metal ion was added individually into the AFSA solution, and the corresponding absorption and emission spectra were documented (Figure 1). The results showed that Cu^2+^ ions triggered a significant fluorescence peak at 766 nm. This increase is ascribed to the coordination of AFSA with Cu^2+^, enhancing the probe’s structural rigidity, impeding the proton transfer process in the excited state, and restraining C=N isomerization (Sun et al., 2023). Conversely, the fluorescence response to other metal ions was insignificant, akin to the blank probe, affirming AFSA’s role as a remarkably sensitive and specific “turn-on” probe for Cu^2+^ ions.

**FIGURE 2:**
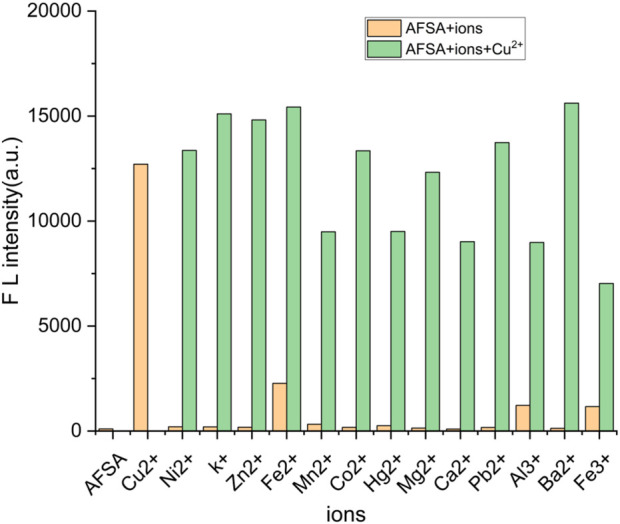
Fluorescence spectra of AFSA (20 μM) solution after adding Cu^2+^ ions (10 μM) upon the addition of various metal ions (10 μM) in EtOH/H_2_O(V/V = 5:1) (λ_ex_ = 384 nm, λ_em_ = 766 nm).

A competitive assay was performed to validate the specificity of fluorescent probe AFSA for detecting Cu^2+^ions and assess the impact of other metal ions on its selectivity. Figure 2 display the absorption and emission data for a mixture of AFSA and Cu^2+^ in the presence of various competing metal ions. Additionally, the influence of common coexisting ions was investigated by adding Cu^2+^ ions (2.5 × 10^−4^ mol/L) into AFSA (5.0 × 10^−5^ mol/L) solutions containing Mg^2+^, Pb^2+^, Zn^2+^, Al^3+^, Mn^2+^, Co^2+^, Cr^3+^, Hg^2+^, Cd^2+^, Fe^2+^, Ni^2+^ and Fe^3+^ (2.5 × 10^−4^ mol/L), individually. The orange bars display the absorbance and intensity reading of probe AFSA with metal ions at 384 nm, while the green bars shows the absorbance and intensity readings of probe AFSA + Cu^2+^ ions with competitive metal ions at 384 nm. The presence of other metal ions did not significantly impact the fluorescence intensity of AFSA with Cu^2+^ (Figure 2). The fluorescence intensity and absorption measurements of AFSA with Cu^2+^ ions were unchanged even with the introduction of additional metal ions. The study demonstrated that the presence of trivalent metals also did not affect the emission of AFSA-Cu^2+^. These results support the use of AFSA as a molecular fluorescent turn-on sensor for Cu^2+^ and its preference for Cu^2+^ ions over other metal ions.

The excellent selectivity and anti-interference performance of AFSA enable accurate and specific recognition of Cu^2+^ in complex actual water samples rich in multiple coexisting metal ions (Na^+^, K^+^, Ca^2+^, Mg^2+^, Fe^3+^), without the need for additional masking agents and simplifying the on-site detection operation.

#### 2.2.2 Quantitative identification of Cu^2+^by probes AFSA

**FIGURE 3:**
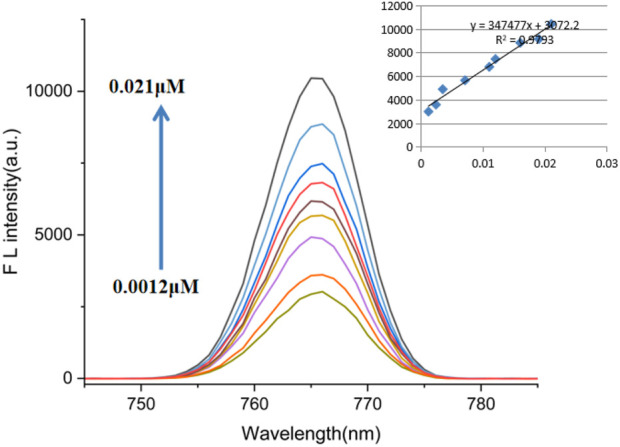
The fluorescence spectra of AFSA (10 μM) with the increasing concentration of Cu^2+^ (0.0012–0.021 μM) in EtOH/H_2_O(V/V = 5/1), (λ_ex_ = 384 nm, λ_em_ = 766 nm).

To investigate the impact of Cu^2+^ addition on the uorescence spectra of probe AFSA, fluorescence spectra of the AFSA probe, experiments were conducted in a solution of EtOH/H_2_O (V/V = 5:1). The fluorescence intensity of AFSA exhibited a gradual increase with rising Cu^2+^ concentration ranging from 0.0012 to 0.021 µM, as depicted in Figure 3. Notably, a good linear relationship was observed between the fluorescence intensity of AFSA probe and Cu^2+^ concentrations within the range of 0.0012–0.021 µM, as shown in Figure 3. This linear correlation underscores the potential of the AFSA probe for precise fluorescence-based quantification of trace Cu^2+^ ions. The calculated detection limits of AFSA were determined to be 0.86 μM, using the formula of limit of detection (LOD, LOD = 3σ/K), based on the fluorescence intensity data.

**FIGURE 4:**
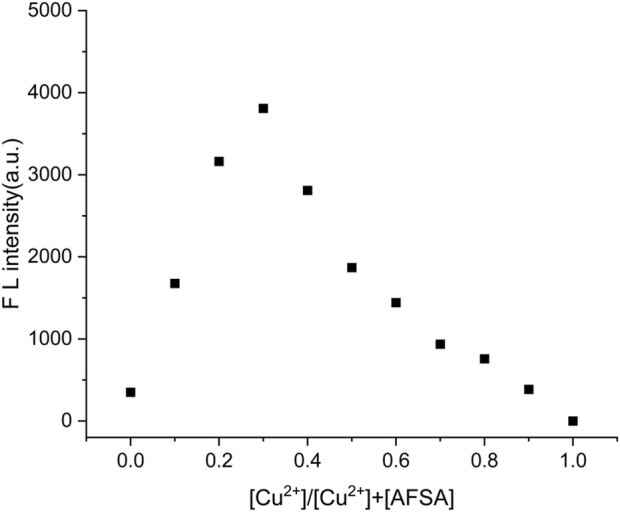
Job’s plot of AFSA and Cu^2+^([AFSA]+[Cu^2+^] = 10 μM) in EtOH/H_2_O(V/V = 5:1) solution by fluorescence spectra, where the fluorscence intensity at 766 nm was plotted againse the mole fraction of [[Cu^2+^]/[[AFSA]+[Cu^2+^]]] (λ_ex_ = 384 nm, λ_em_ = 766 nm).

Furthermore, the interaction mode between AFSA and Cu^2+^ ions was explored through Job’s plot analysis, as shown in Figure 4. To explore the binding ratio of the AFSA probe to Cu^2+^,different ratios of the AFSA probe to Cu^2+^ (0:10,1:9,2:8,3:7,4:6,5:5,6:4,7:3,8:2,9:1,10:0) were prepared. This plot, generated by plotting the molar fraction vs. against the changes in emission intensity at 766 nm, revealed that the fluorescent intensity peaked at a molar fraction of approximately 0.30 (Cu^2+^/Cu^2+^+AFSA). This observation suggests the formation of 1:2 stoichiometric complexes between Cu^2+^ and AFSA. These findings unequivocally demonstrate the potential of the AFSA probe for serving as a high sensitivity and specific fluorescent tool for detecting Cu^2+^ ions.

The good linear response and definite 2:1 binding stoichiometry provide a reliable theoretical and experimental basis for the quantitative detection of trace Cu^2+^, and the detection limit of 0.86 μM is far below the WHO limit for Cu^2+^ in drinking water, fully meeting the requirements of practical environmental monitoring.

#### 2.2.3 Reversibility experiments of AFSA

**FIGURE 5:**
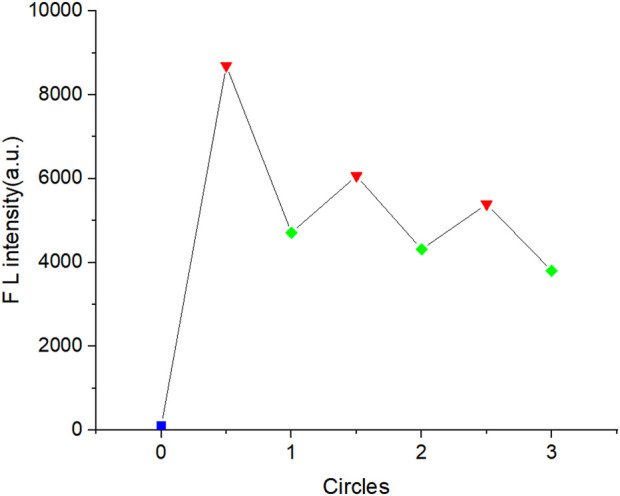
Fluorescent intensity of AFSA (blue plot): AFSA + Cu^2+^(red plot), and AFSA + Cu^2+^+ EDTA (green plot) in EtOH/H_2_O (V/V = 5:1) solution (λ_ex_ = 384 nm, λ_em_ = 766 nm).

The selective recognition of metal ions by probes depends on the binding dynamics between the metal ions and the lone pair electrons of N and O atoms in the probe molecules. To evaluate the recyclability of AFSA, reversibility experiments was performed using EDTA (a Cu^2+^ chelating agent). As shown in Figure 5, AFSA exhibited weak initial fluorescence in the absence of Cu^2+^ ions. Upon Cu^2+^ addition, fluorescence intensity increased singnificantly due to AFSA-Cu^2+^ coordination Subsequent addition of EDTA to the system resulted in a decrease in fluorescence intensity for the AFSA probes, indicating the regeneration of the free AFSA probe. This decrease is a result of the stronger coordination between Cu^2+^ and EDTA, which disrupts the complexation between Cu^2+^ and AFSA. These results clearly illustrate the excellent reversibility of the AFSA probes in detecting Cu^2+^ ions, a critical characteristic for the advancement of devices designed for Cu^2+^ ion sensing applications.

The EDTA-mediated reversible recognition of Cu^2+^ by AFSA allows the probe to be recycled for multiple detection cycles, reducing the consumption of sensing materials and detection costs, and making it suitable for continuous real-time monitoring of Cu^2+^ concentration in environmental water bodies.

#### 2.2.4 Fluorescence enhancement mechanism of AFSA

**SCHEME 2:**
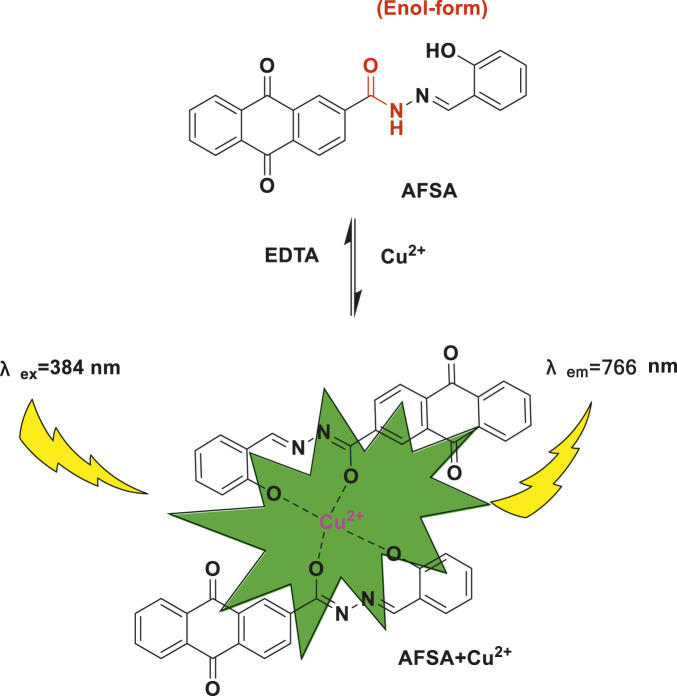
Possible recognition mechanism of probe AFSA to Cu^2+^ ions.

The fluorescence intensity of AFSA at 766 nm gradually increased with increasing Cu^2+^ concentration. Scheme 2 illustrates the complexation mechanism and the underlying reasons for the changes in absorption and fluorescence intensity,and this fluorescence enhancement phenomenon can be explained by several factors. Schiff base fluorescent probes contain conjugated double bonds (-C=N-) that can undergo ketoimine structural isomerization,a process promotes non-radiative transitions in the excited state of the Schiff base, resulting in weak fluorescence. Upon Cu^2+^addition, Cu^2+^ forms complexes with the Schiff base probe, causing deprotonation of the donor molecule and formation of a stable, extended conjugated system. This interaction hinders the structural isomerization of the conjugated double bonds (-C=N-), suppresses the proton transfer process in the excited state of the molecules, and consequently modifies their color and fluorescence properties (O’Boyle et al., 2008; Zhang et al., 2010; Jamroz, 2013). Previous studies have established that the unpaired electrons of nitrogen atoms in the C=N group are involved in coordinating with Cu^2+^ ions, effectively impeding the isomerization process. The interaction between AFSA and Cu^2+^ ions restricts rotation around the C=N bond, thereby preventing C=N isomerization, and this restriction ultimately leads to improved absorption and fluorescence properties (Hanwell et al., 2012; Kar et al., 2007). Analysis of the molecular structure of AFSA reveals that the amide bond in the probe undergoes structural modifications upon binding with Cu^2+^ ions. This interaction induces changes in the system’s energy levels, consequently enhancing fluorescence emission.

#### 2.2.5 Fluorescence responses of probe toward to Cu^2+^

**FIGURE 6:**
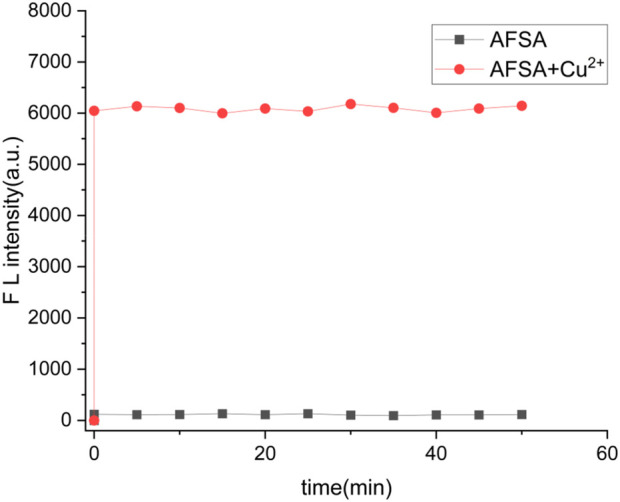
Time-dependent changes of AFSA (10 μM) (black line) with the addition of Cu^2+^ (5 μM) (red line) in EtOH/H_2_O (V/V = 5/1) (λ_ex_ = 384 nm, λ_em_ = 766 nm).

In practical scenarios, the response time of a probe plays a crucial role in assessing its effectiveness. Figure 6 depicts the dynamic changes in fluorescence intensity exhibited by the AFSA probe when exposed to Cu^2+^ ions over time. The experimental results clearly indicate that the interaction between Cu^2+^ ions and the probe reaches completion within a timeframe of less than 1 min. As a result, all fluorescence measurements were consistently performed around 1 min following the introduction of Cu^2+^ ions. The ultra-fast response time of less than 1 min makes AFSA suitable for rapid on-site detection of Cu^2+^, which is superior to traditional instrumental analysis methods with lengthy operation procedures and meets the demand for real-time environmental monitoring.

#### 2.2.6 pH effect of probe AFSA to Cu^2+^

**FIGURE 7:**
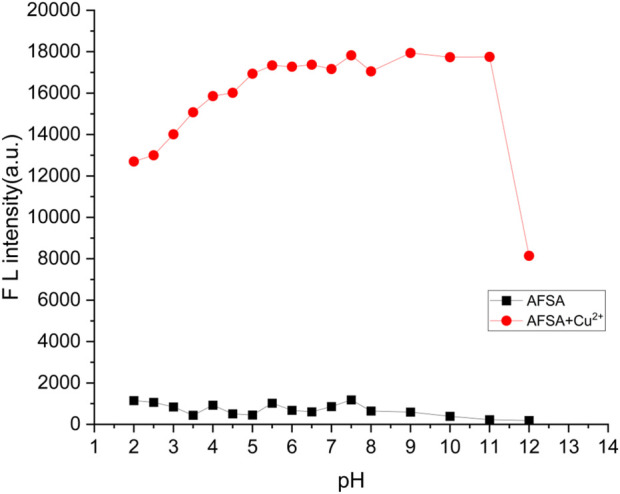
Fluorescence intensity changes of probe AFSA (10 μM) (black line) and AFSA (10 μM)-Cu^2+^ (5 μM) complexes (red line) under different pH values in EtOH/H_2_O (V/V = 5/1) (λ_ex_ = 384 nm, λ_em_ = 766 nm).

To ensure optimal sensing performance under diverse acidity conditions in practical applications, the AFSA probe (2:1 stoichiometry with Cu^2+^) was evaluated in ethanol-water solution (V/V = 5:1), with pH adjusted to 2.0–13.0 (PBS buffer was solely used for pH adjustment in these pH-dependent experiments). As illustrated in Figure 7, the AFSA probe alone exhibited stable fluorescence intensity over pH 2.0–11.0. Upon Cu^2+^ addition (forming the 2:1 AFSA-Cu^2+^ complex), fluorescence intensity increased significantly at pH 2.0–5.0, whereas no obvious fluctuations were observed at pH 5.0–11.0. This observation is likely due to Cu^2+^ hydrolysis under acidic conditions, which impedes AFSA-Cu^2+^ complex formation. Collectively, the as-constructed AFSA probe acts as a high-performance fluorescent sensor, effective over a broad pH range of 2.0–11.0 and particularly applicable for detection at pH 5.0–11.0. The stable fluorescence response of AFSA toward Cu^2+^ in the wide pH range of 5.0–11.0 enables its adaptation to the natural pH variation of environmental water samples (e.g., tap water, surface water), expanding its practical application scope and avoiding the need for pre-adjustment of sample pH.

Compared with previously reported Cu^2+^ probes, AFSA presents prominent comprehensive performance advantages. First, it has a broad pH working range of 5.0–11.0, outperforming most probes that only maintain stable performance in the 6.0–9.0 pH interval. Second, it displays near-infrared fluorescence emission at 766 nm, which effectively mitigates background fluorescence interference in practical detection. This study also objectively notes that the detection limit of AFSA for Cu^2+^ is 0.86 μM, a moderate level among analogous probes. Nevertheless, this value is far below the 20 μM regulatory limit for Cu^2+^ in drinking water set by the World Health Organization, and thus fully satisfies the practical demands for on-site rapid detection (He et al., 2019; Liu et al., 2023; Ma et al., 2025; Moghadam et al., 2019; Padhan et al., 2019; Sun et al., 2023), as summarized in Table 1.

**TABLE 1:** Comparison of some fluorescent probes for Cu^2+^ detection.

Structure of probes	Tested media	pH	LOD	Modes (Probe:Cu^2+^)	Ref.
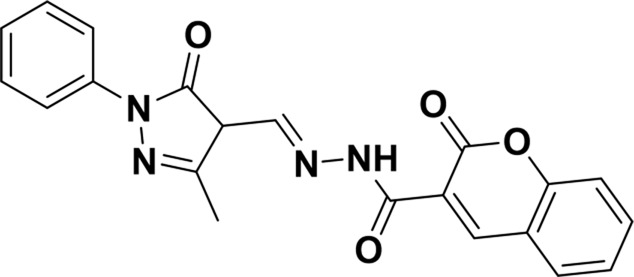	EtOH/H_2_O (1: 1, V/V)	4.0–11.0	15.16 nM	1:1	Liu et al. (2023) Our previous work
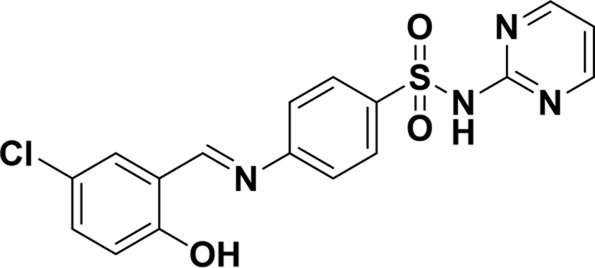	DMSO/H_2_O V/V = 3:2	6.0–10.0	3.98 × 10^−8^ M	2:1	Sun et al. (2023)
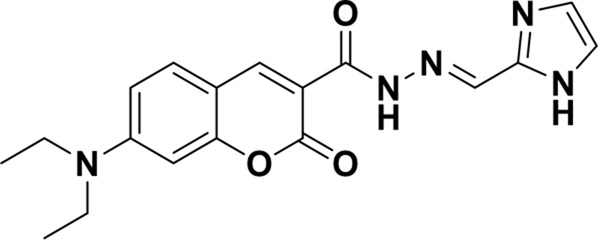	CH_3_CN:HEPES (3:2, V/V)	—	0.12 μM	1:1	He et al. (2019)
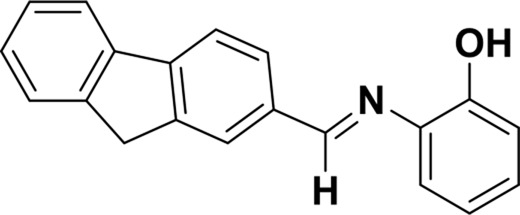	CH_3_CN solution	—	1.54 × 10^−9^ M	1:1	Moghadam et al. (2019)
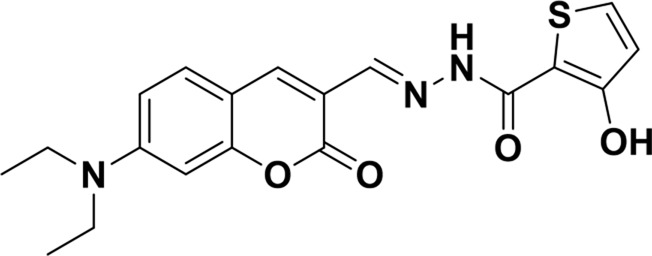	THF/H_2_O	3.0–8.0	1.76 × 10^−7^ M	1:1	Ma et al. (2025)
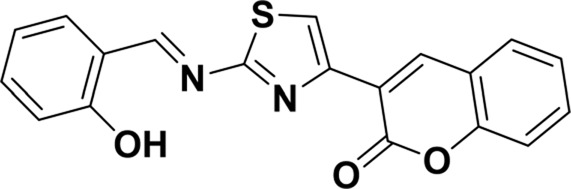	DMSO–water	5.0–9.0	24 nM	1:1	Padhan et al. (2019)
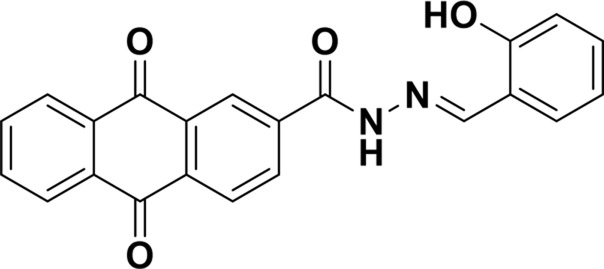	EtOH/H_2_O V/V = 5:1	5.0–11.0	0.86 μM	2:1	Tihs work

#### 2.2.7 Geometry optimization

**FIGURE 8:**
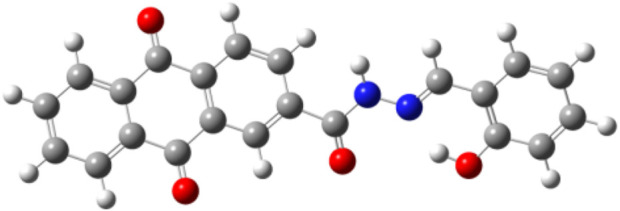
Optimized geometry of AFSA.

The Schiff base AFSA was structurally optimized using density functional theory (DFT) methods (Afsan et al., 2020). Ground-state geometry optimization was performed with Gaussian 09 software to obtain the minimum-energy conformer. The absence of imaginary frequencies in the DFT calculations confirmed the stability of the optimized structure. Figure 8 displays the final geometry, with key bond parameters compared against both theoretical and experimental literature data. In the benzene ring of AFSA, the C–C bond lengths typically range around 1.395 Å (Shan et al., 2025), while the C=O bond length was experimentally determined as 1.224 Å, closely matching the DFT-derived value of 1.227 Å (Khamees et al., 2018). For AFSA, the computed C–C bond lengths (1.382–1.40 Å) and C=O bond length (1.223 Å) align well with these references. Similarly, the thiodiazole moiety exhibits N=C bond lengths of 1.283–1.340 Å experimentally, consistent with DFT-calculated values (1.296–1.371 Å). Reported N–N bond lengths span 1.363–1.391 Å (experimental) and 1.370–1.61 Å (theoretical) (Ati et al., 2024; Wang and Zhao, 2023). In agreement with our computed ranges (1.301–1.306 Å for N=C and 1.356–1.795 Å for N–N). The strong correlation between our DFT-derived geometry and published data validates the optimization process. The final structure was subsequently employed for further computational analyses. The good consistency between the DFT-optimized geometric parameters and the reported literature data validates the reliability of the molecular model, providing an accurate structural basis for the subsequent theoretical analysis of the photophysical properties and Cu^2+^ recognition mechanism of AFSA.

#### 2.2.8 Frontier molecular orbital (FMO) analysis

**FIGURE 9:**
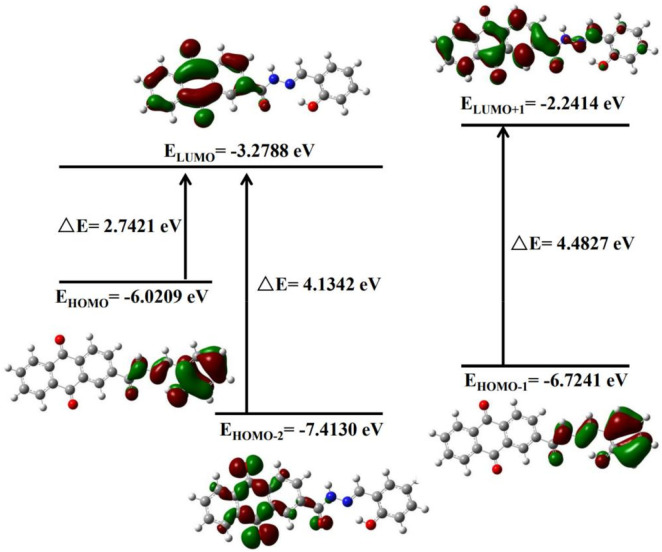
Frontier molecular orbitals with energy gap for AFSA.

**FIGURE 10:**
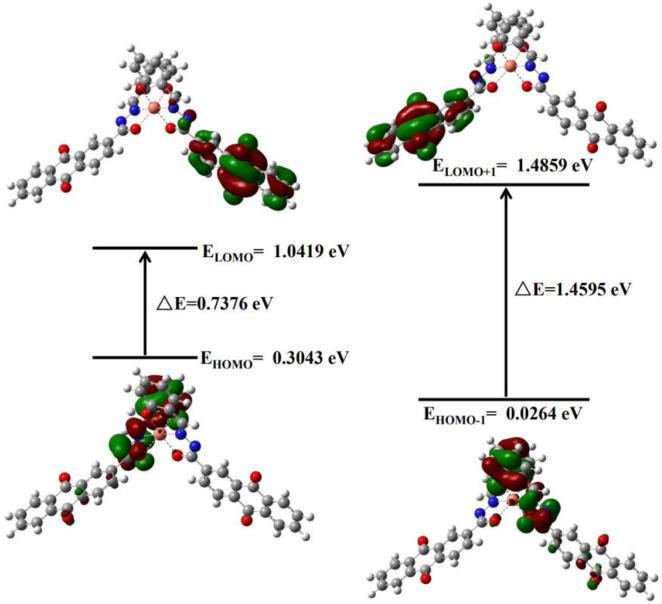
Frontier molecular orbitals with energy gap for AFSA + Cu^2+^.

FMO analysis was conducted at the CAM-B3LYP level, it aimed to clarify the electrochemical properties of the AFSA probe and its AFSA-Cu^2+^ complex. The calculation determined the lowest unoccupied molecular orbital (ELUMO) and highest occupied molecular orbital (EHOMO) energy levels, as well as the energy gap (ΔE). ΔE is a key indicator of molecule reactivity and stability. EHOMO and ELUMO reflect electron-donating and electron-accepting abilites, respectively. A lower ELUMO means stronger electron-accepting capacity, whereas a higher EHOMO indicates greater capacity to electrons-donating. FMO plots of AFSA and AFSA-Cu^2+^ complex are persented in Figures 9, 10, respectively. The AFSA ligand has a HOMO-LUMO gap of 2.7421 eV. Coordination with Cu^2+^ reduces this gap to 0.7373 eV, indicating altered electronic structure. For AFSA, HOMO is localized on hydrazide and anthraquinone groups. LUMO is mainly on the anthraquinone moiety, suggesting electron transfer from anthraquinone to hydrazide. In the AFSA-Cu^2+^ complex, both HUMO and LUMO are concentrated around the Cu^2+^ ion, and two AFSA ligands coordinate with one Cu^2+^ ion. Coordination sites are hydroxyl oxygen atom of salicylaldehyde and imine nitrogen atoms of the “C=N groups” in the AFSA molecules,the AFSA molecule exhibits structural mismatches along its conjugated backbone.

Following coordination with Cu^2+^, the energies of the LUMO and HOMO of the AFSA-Cu^2+^ complex are determined to be 1.0419 eV and 0.3043 eV, respectively, resulting in an energy gap of 1.4595 eV. The electron cloud distribution is predominantly concentrated on the imine bond, while the electron density is also notably focused on the entire salicylaldehyde framework along with the “C=N groups” (Yue et al., 2022). The comparison of the energy gap between the AFSA-Cu^2+^ system and the pristine AFSA molecule suggests that the addition of Cu^2+^ enhances the stability of the system. The decrease in the ΔE value indicates the establishment of a stable coordination environment between the AFSA probe and Cu^2+^ ions. This is consistent with the results in Section 2.2.6.

FMO analysis clarifies the coordination sites and electron transfer mechanism of AFSA with Cu^2+^ at the molecular level, providing a rigorous theoretical explanation for the “turn-on” fluorescence response of the probe and offering a valuable design strategy for the structural modification of anthraquinone-based Schiff base fluorescent probes for heavy metal ion detection.

## 3 Materials and methods

### 3.1 Materials and instruments

All chemical reagents were purchased from Macklin Co., Ltd. (Shanghai, China) and can be used without purification. Melting points were determined on an Electrothermal 9100 melting point apparatus without corrected. NMR spectroscopy was recorded on AVANCE 500 MHz or 600 MHz (Bruker, Karlsruhe, Germany) instrument. Electrospray ionization mass spectrometry (ESI-MS) was measured by RF-6000 luminescence spectrophotometer (Bruker, Karlsruhe, Germany). ESI-MS data was recorded with Mariner System 5,304 mass spectrometer. Fluorescence spectral data were collected using a Horiba FluoroMax-4 spectrophotometer. Elemental analyses (C, H, and N) were gathered on a CHN-O-Rapid instrument within 0.4% of the theoretical values.

### 3.2 Synthesis of probe AFSA

#### 3.2.1 Synthesis of 9,10-anthraquinone-2-carboxylic acetate (2)

Compound 1 (7.4 mmol) and SOCl_2_ (8.9 mmol) were mixed and refluxed at 78 °C for 2 h, the overdose of SOCl_2_ was removed in vacuum and excess of methanol was added to the residue and stirred overnight at 25 °C, the precipitate was filtered and washed to obtain a light yellow solid with the yield of 92.01%. mp.214 °C–217 °C. ^1^H NMR (500 MHz, DMSO-*d*_*6*_) δ 8.59 (s, 1H), 8.36 (d, J = 7.4 Hz, 1H), 8.27 (d, J = 7.6 Hz, 1H), 8.20 (s, 2H), 7.95 (s, 2H), 3.95 (s, 3H). MS (ESI): 267.08 (M + H)^+^.Anal.Calcd for C_16_H_10_O_4_: C, 72.18; H, 3.79. Found: C, 72.03; H, 3.68.

#### 3.2.2 Synthesis of 9,10-anthraquinone-2-carbohydrazide (3)

Compound 2 (8.57 mmol) and 85% hydrazine hydrate (8.57 mmol) were mixed in 50 mL ethanol was stirred at 25 °C for 8 h. Then, the liquid was dumped into crude ice, and then the precipitated solid was collected and washed by cold ethanol and water three times to obtain compound 3, yielding 82.55%, used in the next step without further purified (Alam and Khan, 2023).

#### 3.2.3 Synthesis of AFSA

Compound 3 (10 mmol) and salicylaldehyde (10 mmol) were dissolved in 20 mL ethanol with two drops of acetic acid as a catalytic agent and stirred at 78 °C for 4.5 h. Then, the solution was cooled to 0 °C, and the precipitated solid was collected and washed with cooled ethanol to give lightly yellow solid compound (AFSA) with a yield of 61.82%. ^1^H NMR (600 MHz, DMSO-*d*_*6*_) δ 12.61 (s, 1H), 11.28 (s, 1H), 8.75 (d, J = 5.0 Hz, 2H), 8.67 (s, 1H), 8.44 (d, J = 7.6 Hz, 1H), 8.37 (d, J = 7.6 Hz, 1H), 8.31 (d, J = 7.9 Hz, 1H), 8.22 (d, J = 13.4 Hz, 2H), 8.19–8.12 (m, 1H), 7.94 (s, 2H), 7.91 (d, J = 2.8 Hz, 1H), 7.59 (s, 1H), 7.31 (t, J = 7.0 Hz, 1H), 7.00–6.90 (m, 2H). ^13^C NMR (151 MHz, DMSO-*d*_*6*_) δ 182.93, 182.49, 168.67, 161.96, 158.33, 150.32, 138.52, 135.48, 135.09, 134.88, 133.67, 133.22, 131.99, 130.20, 128.24, 127.81, 127.78, 127.70, 127.35, 127.07, 127.07,126.97, 126.67, 119.47, 119.25,118.53, 117.07. MS (ESI): 371.10 (M + H)^+^.Anal.Calcd for C_22_H_14_N_2_O_4_: C,71.35; H, 3.81. Found: C, 71.55; H, 3.92.

### 3.3 General procedure for the spectrum measurement

The stock solution of AFSA at a concentration of 0.1 mM was meticulously prepared by dissolving it in a solvent mixture EtOH and water (V/V = 5:1). Individual metal ions, including copper (Cu^2+^), iron (Fe^3+^), aluminum (Al^3+^), magnesium (Mg^2+^), and calcium (Ca^2+^) were each prepared as 0.1 mM solutions in water. The Cu^2+^ solution was freshly prepared using copper sulfate pentahydrate (CuSO_4_·5H_2_O) just before its intended use. Solutions of Fe^3+^, Al^3+^, Mg^2+^, Ca^2+^, and Cr^3+^ were derived from their respective chloride salts. On the other hand, solutions of cobalt (Co^2+^), nickel (Ni^2+^), ferrous iron (Fe^2+^), zinc (Zn^2+^), mercury (Hg^2+^), manganese (Mn^2+^), and lead (Pb^2+^) were prepared from perchlorates.

To conduct the experiments, various concentrations of these metal ions were introduced into a phosphate-buffered saline (PBS) solution (2.0 mL, 0.1 M, pH = 7.4) along with compound AFSA (1.0 mL, 0.1 mM) in a 10 mL color comparison tube. Following dilution with doubly distilled water, the resulting mixture was allowed to equilibrate for 10 min before measurements were taken. Excitation for all fluorescence studies was set at 384 nm. The fluorescence measurements were performed using a RF-6000 luminescence spectrophotometer, which was equipped with a xenon lamp and a 1.0 cm quartz cuvette, all maintained at room temperature. Moreover, NMR data was acquired utilizing on AVANCE 500 MHz or 600 MHz (Bruker, Karlsruhe, Germany) instrument, with chemical shifts reported in parts per million (ppm) relative to tetramethylsilane (TMS) as the internal standard.

### 3.4 Computational

All the computational investigations were executed with the aid of the Gaussian 9.0 program (Sun et al., 2023). Initial molecular geometries of the probe (AFSA) and its complexes were optimized using the molecular modeling software Avogadro (Hanwell et al., 2012). For the free AFSA molecule, density functional theory (DFT) calculations were conducted at the B3LYP (Zhang et al., 2010) level with the 6–311++G (d,p) basis set, while the LANL2DZ pseudopotential basis set was employed for the AFSA-Cu^2+^ complex to account for relativistic effects in the transition metal. Vibrational frequency assignments for AFSA were systematically analyzed using the VEDA4 program (Jamroz, 2013), which facilitated the interpretation of infrared spectra through normal mode decomposition. A detailed breakdown of the electronic transitions contributing to each absorption band was obtained using the GaussSum 3.0 package (O’Boyle et al., 2008), which provided orbital contributions and excitation characterizations. Additionally, the relative quantum yield and fluorescence lifetime of AFSA were quantitatively assessed using FluorTools (Khamees et al., 2018) and PhotochemCAD (Ati et al., 2024), respectively. These analyses ensured a comprehensive understanding of the probe’s photophysical properties under varying conditions.

## 4 Conclusion

A novel anthraquinone-based Schiff base turn-on fluorescent probe AFSA was successfully synthesized for the selective and sensitive detection of Cu^2+^ ions, and its sensing performance was systematically investigated in an EtOH/H_2_O (5:1, V/V) binary system. Multiple spectroscopic characterization techniques verified the structural integrity of AFSA, and Job’s plot analysis confirmed the formation of a 2:1 stoichiometric complex between AFSA and Cu^2+^, with a detection limit of 0.86 μM for Cu^2+^. The probe exhibited excellent comprehensive sensing properties, including ultrahigh selectivity and anti-interference ability against common coexisting metal ions, an ultrafast response time of less than 1 min for Cu^2+^ recognition, stable fluorescence emission over a wide pH range of 5.0–11.0, and good EDTA-mediated reversible recognition performance. Density functional theory (DFT) and frontier molecular orbital (FMO) calculations further elucidated the structural basis and electron transfer mechanism of the probe’s fluorescence response to Cu^2+^ at the molecular level, providing a rigorous theoretical explanation for the experimental results of the turn-on fluorescence behavior.

The distinctive performance characteristics of AFSA render it a promising fluorescent sensing material for the on-site rapid monitoring of Cu^2+^ in environmental water samples (e.g., tap water, surface water). Its detection limit is far below the 20 μM regulatory limit for Cu^2+^ in drinking water specified by the World Health Organization, fully meeting the practical requirements of environmental water quality monitoring; the wide pH adaptability allows direct application to natural water samples without pre-adjustment of pH, simplifying the pre-treatment process of actual samples. The anthraquinone-Schiff base molecular design strategy developed in this work also provides a valuable reference for the construction of novel fluorescent probes for heavy metal ion detection. Future research will focus on the structural modification of AFSA to further improve its detection sensitivity for trace Cu^2+^ in complex water bodies, and the combination of AFSA with solid-phase carrier materials to develop portable detection devices, thereby expanding its practical application potential in the field of environmental monitoring.

## Data Availability

The raw data supporting the conclusions of this article will be made available by the authors, without undue reservation.

## References

[B1] Afsan Z. Roisnel T. Tabassum S. Arjmand F. (2020). Structure elucidation spectroscopic, single crystal X-ray diffraction and computational DFT studies of new tailored benzenesulfonamide derived schiff base copper(II) intercalating complexes: comprehensive biological profile DNA binding, pBR322 DNA cleavage, topo I inhibition and cytotoxic activity. Bioorg Chem. 94, 103427. 10.1016/j.bioorg.2019.103427 31735357

[B2] Alam M. Z. Khan S. A. (2023). A review on schiff base as a versatile fluorescent chemo-sensors tool for detection of Cu and Fe metal ion. J. Fluoresc. 33, 1241–1272. 10.1007/s10895-022-03102-1 36708420

[B3] Albertos P. Sanchez-Vicente I. Franco J. M. Solano R. Gerna D. Roach T. (2022). Selenbp1 and Semo-1: copper-dependent H2s-Generating enzymes in humans and in the model organism C. elegans. Free Radic. Biol. Med. 189, 22.

[B4] Ati G. A. Chkirate K. El-Guourrami O. Chakchak H. Tüzün B. Mague J. T. (2024). Schiff base compounds constructed from pyrazole–acetamide: synthesis, spectroscopic characterization, crystal structure, DFT, molecular docking and antioxidant activity. J. Mol. Struct. 1295, 136637. 10.1016/j.molstruc.2023.136637

[B5] Brewer G. J. (2010). Copper toxicity in the general population. Clin. Neurophysiol. 121, 459–460. 10.1016/j.clinph.2009.12.015 20071223

[B6] Chellan P. Sadler P. J. (2015). The elements of life and medicines. Philosophical Trans. R. Soc. A Math. Phys. and Eng. Sci. 373, 20140182. 10.1098/rsta.2014.0182 25666066 PMC4342972

[B7] Chen J. M. Luo R. Q. Li S. Shao J. P. Wang T. Xie S. M. (2024). A novel NIR fluorescent probe for copper(II) imaging in parkinson's disease mouse brain. Chem. Sci. 15, 13082–13089. 10.1039/d4sc03445g 39148792 PMC11323298

[B8] Dujols V. Ford F. Czarnik A. W. (1997). A long-wavelength fluorescent chemodosimeter selective for Cu(II) ion in water. J. Am. Chem. Soc. 119, 7386–7387. 10.1021/ja971221g

[B9] Golbedaghi R. Justino L. L. G. Ooshall F. Jamehbozorgi S. Abdolmaleki M. Fausto R. (2021). A new schiff base ligand as a fluorescence probe for Cu(II) detection in semi-aqueous solution: synthesis, characterization, fluorescence and mechanistic insight. Inorganica Chim. Acta 528, 120623. 10.1016/j.ica.2021.120623

[B10] Gumpu M. B. Sethuraman S. Krishnan U. M. Rayappan J. B. B. (2015). A review on detection of heavy metal ions in water - an electrochemical approach. Sensors Actuators B-Chemical 213, 515–533. 10.1016/j.snb.2015.02.122

[B11] Hanwell M. D. Curtis D. E. Lonie D. C. Vandermeersch T. Zurek E. Hutchison G. R. (2012). Avogadro: an advanced semantic chemical editor, visualization, and analysis platform. J. Cheminform 4, 17. 10.1186/1758-2946-4-17 22889332 PMC3542060

[B12] He G. Hua X. Yang N. Li L. Xu J. Yang L. (2019). Synthesis and application of a “turn on” fluorescent probe for glutathione based on a copper complex of coumarin hydrazide schiff base derivative. Bioorg. Chem. 91, 103176. 10.1016/j.bioorg.2019.103176 31404797

[B13] Jamroz M. H. (2013). Vibrational energy distribution analysis (VEDA): scopes and limitations. Spectrochim. Acta A Mol. Biomol. Spectrosc. 114, 220–230. 10.1016/j.saa.2013.05.096 23778167

[B14] Ji L. G. Fu Y. T. Yang N. Wang M. F. Yang L. L. Wang Q. Z. (2022). A fluorescence “turn-on” probe for Cu (II) based on flavonoid intermediates generated by copper-induced oxidative cyclization and its fluorescence imaging in living cells. Anal. Biochem. 655, 114855. 10.1016/j.ab.2022.114855 35987417

[B15] Kar P. Suresh M. Kumar D. K. Jose D. A. Ganguly B. Das A. (2007). Preferential binding of the magnesium ion by anthraquinone based chromogenic receptors. Polyhedron 26, 1317–1322. 10.1016/j.poly.2006.10.051

[B16] Khamees H. A. Jyothi M. Khanum S. A. Madegowda M. (2018). Synthesis, crystal structure, spectroscopic characterization, docking simulation and density functional studies of 1-(3,4-dimethoxyphenyl) -3-(4-flurophenyl)-propan-1-one. J. Mol. Struct. 1161, 199–217. 10.1016/j.molstruc.2018.02.045

[B17] Kumari N. Singh S. Baral M. Kanungo B. K. (2023). Schiff bases: a versatile fluorescence probe in sensing cations. J. Fluoresc. 33, 859–893. 10.1007/s10895-022-03135-6 36633727

[B18] Li B. Y. Yang R. J. Chen X. J. He J. Y. Lu Z. J. Li Y. S. (2024). Copper mitigates atrazine-induced neurotoxicity in parkinson's disease models. Mol. Neurobiol. 62 (4), 5202–5215. 10.1007/s12035-024-04609-3 39527184

[B19] Liang Z. Q. Song D. D. Li Z. C. Xu S. H. Dai G. L. Ye C. Q. (2024). Bright photoactivatable probes based on triphenylethylene for Cu detection in tap water and tea samples. Food Chem. 434. 10.1016/j.foodchem.2023.137439 37729781

[B20] Liu J. Cheng P.-Y. Chen S. Wang M. Wei K. Li Y. (2023). Preparation and application of a fast, naked-eye, highly selective, and highly sensitive fluorescent probe of schiff base for detection of Cu^2+^ . Chemosensors 11, 556. 10.3390/chemosensors11110556

[B21] Ma Q. Yang X. Zhao Y. (2025). Development of a coumarin-based schiff base fluorescent probe and its application in detection of Cu^2+^ . J. Fluoresc. 35, 7573–7584. 10.1007/s10895-024-04114-9 39776091

[B22] Mashhadizadeh M. H. Zadmehr M. R. Allah-Abadi H. (2006). Selective preconcentration and solid phase extraction of ultra trace copper(II) from natural water and human hair and determination by atomic absorption spectroscopy. Asian J. Chem. 18, 137–144.

[B23] Moghadam F. N. Amirnasr M. Meghdadi S. Eskandari K. Buchholz A. Plass W. (2019). A new fluorene derived schiff-base as a dual selective fluorescent probe for Cu and CN. Spectrochimica Acta Part A-Molecular Biomol. Spectrosc. 207, 6–15. 10.1016/j.saa.2018.08.058 30195186

[B24] Moghaddam M. R. Carrara S. Hogan C. F. (2019). Multi-colour bipolar electrochemiluminescence for heavy metal ion detection. Chem. Commun. 55, 1024–1027. 10.1039/c8cc08472f 30480267

[B25] O'boyle N. M. Tenderholt A. L. Langner K. M. (2008). Cclib: a library for package-independent computational chemistry algorithms. J. Comput. Chem. 29, 839–845. 10.1002/jcc.20823 17849392

[B26] Padhan S. K. Murmu N. Mahapatra S. Dalai M. K. Sahu S. N. (2019). Ultrasensitive detection of aqueous Cu ions by a coumarin-salicylidene based AIEgen. Mater. Chem. Front. 3, 2437–2447. 10.1039/c9qm00394k

[B27] Quintanar L. (2024). Copper redox chemistry in degenerative diseases: from alzheimer's to cataract disease. Free Radic. Biol. Med. 224, S5. 10.1016/j.freeradbiomed.2024.10.267

[B28] Shan S.-J. Cao Y.-Y. Yao P. Du P.-P. Chen S. Li H.-L. (2025). Crystal structure of 1,3-dihydroxy-6,8-dimethoxy-2-(6-methyltetrahydro-2Hpyran-2-yl)-4a,9a-dihydroanthracene-9,10-dione, C_22_H_22_O_7_ . Z. für Kristallogr. - New Cryst. Struct. 240 (2), 233–235. 10.1515/ncrs-2024-0441

[B29] Sun Y.-X. Sun Y.-G. Deng Z.-P. Jia Y.-H. Han W.-Y. Wang J.-J. (2023). A turn-off fluorescent probe for the detection of copper(II) ion based on a salicylaldehyde derivatives Schiff-base. J. Mol. Struct. 1291, 136069. 10.1016/j.molstruc.2023.136069

[B30] Teschke R. Eickhoff A. (2024). Wilson disease: copper-mediated cuproptosis, iron-related ferroptosis, and clinical highlights, with comprehensive and critical analysis update. Int. J. Mol. Sci. 25, 4753. 10.3390/ijms25094753 38731973 PMC11084815

[B31] Uauy R. Olivares M. Gonzalez M. (1998). Essentiality of copper in humans. Am. J. Clin. Nutr. 67, 952s–959s. 10.1093/ajcn/67.5.952S 9587135

[B32] Udhayakumari D. Velmathi S. Sung Y. M. Wu S. P. (2014). Highly fluorescent probe for copper (II) ion based on commercially available compounds and live cell imaging. Sensors Actuators B-Chemical 198, 285–293. 10.1016/j.snb.2014.03.063

[B33] Wang Y. Zhao C. S. (2023). Synthesis, crystal structure and DFT study of ethyl 5-(Trimethylsilyl)-1-1H-Pyrazole-3-Carboxylate. J. Struct. Chem. 64, 603–617. 10.1134/s002247662304008x

[B34] Wang Z. R. Zhang T. J. Wang Q. Y. Xu E. Y. Zhang X. Zhang Z. H. (2023). (E)-2-styrylanthracene-9,10-dione derivatives as novel fluorescent probes: synthesis, photophysical properties and application in mitochondria imaging. Spectrochimica Acta Part A-Molecular Biomol. Spectrosc. 286, 121988. 10.1016/j.saa.2022.121988 36308828

[B35] Yue Y. N. La Y. T. Zhang J. Dong W. K. (2022). Synthesis, crystal structure, fluorescence properties and theoretical calculations of heterobimetallic 3d-4f complex with a flexible bis(salamo)-type ligand. J. Mol. Struct. 1264, 133272. 10.1016/j.molstruc.2022.133272

[B36] Zeng X. D. Gao S. Jiang C. Duan Q. X. Ma M. S. Liu Z. G. (2021). Rhodol-derived turn-on fluorescent probe for copper ions with high selectivity and sensitivity. Luminescence 36, 1761–1766. 10.1002/bio.4118 34250703

[B37] Zhang I. Y. Wu J. Xu X. (2010). Extending the reliability and applicability of B3LYP. Chem. Commun. (Camb) 46, 3057–3070. 10.1039/c000677g 20372746

[B38] Zhang T. Li W. D. Li X. Peng Y. D. Dong W. K. (2022). Unusual fluorescence behavior of first 3d-3d' heterobimetallic [Cu(II)2Mn(II)] complex bearing a bis(salamo)-based ligand. J. Mol. Struct. 1260, 132854. 10.1016/j.molstruc.2022.132854

[B39] Zhao C. Lu W. J. Aziz A. Asif M. Dong C. Shuang S. M. (2025). A neoteric smartphone-assisted colorimetric and fluorometric dual-mode probe based on anthraquinone derived schiff base for wide-range pH and Cu^2+^/S^2-^determination in bioimaging. Biosens. and Bioelectron. 271, 117085. 10.1016/j.bios.2024.117085 39721463

[B40] Zhou W. J. Hu Z. Wei J. X. Lu H. X. Dai H. Q. Zhao J. C. (2022). A ratiometric fluorescent probe based on PCN-224 for rapid and ultrasensitive detection of copper ions. Compos. Commun. 33, 101221. 10.1016/j.coco.2022.101221

[B41] Zhu S. Z. Zhu S. R. Xing F. F. (2022). Anthraquinone-1,8-Derived (Pseudo-) crown and lariat ethers:design and applications as fluorescent and chromogenic ion (pair) sensors. Chemistry-an Asian J. 17, e202200564. 10.1002/asia.202200564 35763343

[B42] Zhu J. P. Graziotto M. E. Cottam V. Hawtrey T. Adair L. D. Trist B. G. (2024). Near-infrared ratiometric fluorescent probe for detecting endogenous Cu in the brain. Acs Sensors 9, 2858–2868. 10.1021/acssensors.3c02549 38787339

