# Supplementary material for: Anthraquinone-based turn-on fluorescence probe for selective and sensitive detection of Cu2+ ions
Source: Front Chem. 2026 Apr 14;14:1761329. doi: 10.3389/fchem.2026.1761329 (PMC13123470; doi:10.3389/fchem.2026.1761329)
Supplement: Supplementary file 1 [file DataSheet1.docx]

Supplementary Material

**Anthraquinone-based Turn-On Fluorescence Probe for Selective and Susceptible Detection of Cu^2+^ Ions**

Jiang-song Jia^1†^,Hong-Lei Li^2†^,Yi-fan Xu^2^, Wen-ming Zhao^1^ and Jun Sun^1^*

^1^Department of Pharmacy, Henan Provincial People's Hospital; People's Hospital of Zhengzhou University; People's Hospital of Henan University, Zhengzhou, 450003, China;

^2^Department of Pharmacy, Kangda College of Nanjing Medical University, Lianyungang, 222000, China;

*Correspondence:SJun@zzu.edu.cn

† These authors contributed equally to this work.

**Table of Contents**

**Copies of ^1^H and ^13^C NMR spectra of AFSA………..............…........................……………...S2**

**S1**

*(E)-N'-(2-hydroxybenzylidene)-9,10-dioxo-9,10-dihydroanthracene-2-carbohydrazide* (**AFSA**)

**S2**
